# Terbinafine Resistant Trichophyton Indotineae in Sweden

**DOI:** 10.2340/actadv.v105.42089

**Published:** 2025-02-10

**Authors:** Sahel MOHSENI, Nissrine ABOU-CHAKRA, Karl OLDBERG, Erja CHRYSSANTHOU, Ewa YOUNG

**Affiliations:** 1 Department of Dermatology and Venereology, Lund University, Skåne University Hospital, Sweden; 2 Unit of Mycology, Statens Serum Institut, Copenhagen, Denmark; 3 Department of Clinical Microbiology, Infection Prevention and Control, Office for Medical Services, Region Skåne, Sweden; 4 Department of Clinical Sciences Lund, Division of Infection Medicine, Lund University, Lund, Sweden; 5 Department of Clinical Microbiology, Karolinska University Laboratory, Stockholm, Sweden

For more than a decade, South Asia has witnessed an endemic surge of terbinafine resistant dermatophyte infections that pose challenges to treatment, primarily associated with *Trichophyton indotineae* (1). This emerging fungal species primarily spreads through person-to-person contact, resulting in widespread inflammatory and pruritic dermatophytoses, predominantly affecting the groin, genitals, trunk, and extremities. Formerly identified as *Trichophyton mentagrophytes genotype VIII* during its initial emergence in India, it has since been reclassified into its own distinct species, *Trichophyton indotineae* (1, 2). This pathogen has extended its reach beyond Asia, with instances of this strain being identified in multiple European countries such as Denmark and the UK, Middle East, North America, and Australia (3–5). This is the first report of *Trichophyton indotineae* cases in Sweden.

Most strains of *Trichophyton indotineae* are resistant to terbinafine, which is commonly used in topical or systemic therapy of dermatophytosis. The mechanism of resistance to this agent is caused by point mutations in the squalene epoxidase gene, causing squalene accumulation and depletion of ergosterol, thereby compromising the integrity of the fungal plasma membrane (6). As *Trichophyton indotineae* continues to spread, there is a growing need for international collaboration in research, surveillance, and public health initiatives to mitigate its impact.

Until now 51 cases of *T. indotineae* infection in Sweden have been identified at Karolinska University Laboratory in Stockholm during 2023–2024. Below, 3 of the Swedish cases are presented, detailing their clinical picture, methods of species identification, and treatment.

## CASES

*Patient A.* A healthy 21-year-old woman developed a widespread pruritic erythematous plaque on her thighs during the summer of 2021 after visiting relatives in Lebanon. She received fluconazole treatment at a dosage of 150 mg once a week for 4 weeks from her general practitioner. There was no improvement during treatment, and the erythema continued to spread. She underwent an initial dermatologic evaluation in February 2022. Examination noted large, annular, scaly, pruritic plaques on her thighs and breast. A diagnosis of tinea was made, and she commenced oral terbinafine therapy at a dosage of 250 mg twice daily for 4 weeks. Fungal culture revealed the presence of *Trichophyton mentagrophytes* complex.

No improvement was noted during the follow-up, leading to a new sample and an extension of terbinafine treatment to 8 weeks in total with minimal improvement. The isolate was sent to the reference laboratory at Karolinska University Laboratory for antifungal susceptibility testing, showing resistance to terbinafine (minimum inhibitory concentration [MIC] > 8 mg/mL) and susceptibility to itraconazole.

Consequently, itraconazole therapy was initiated at a dosage of 100 mg daily for 4 weeks. The lesions completely resolved after the course of itraconazole treatment, and no recurrence was observed after 6 months. The isolate was later re-analyzed and identified as *T. indotineae.*

**Fig. 1:**
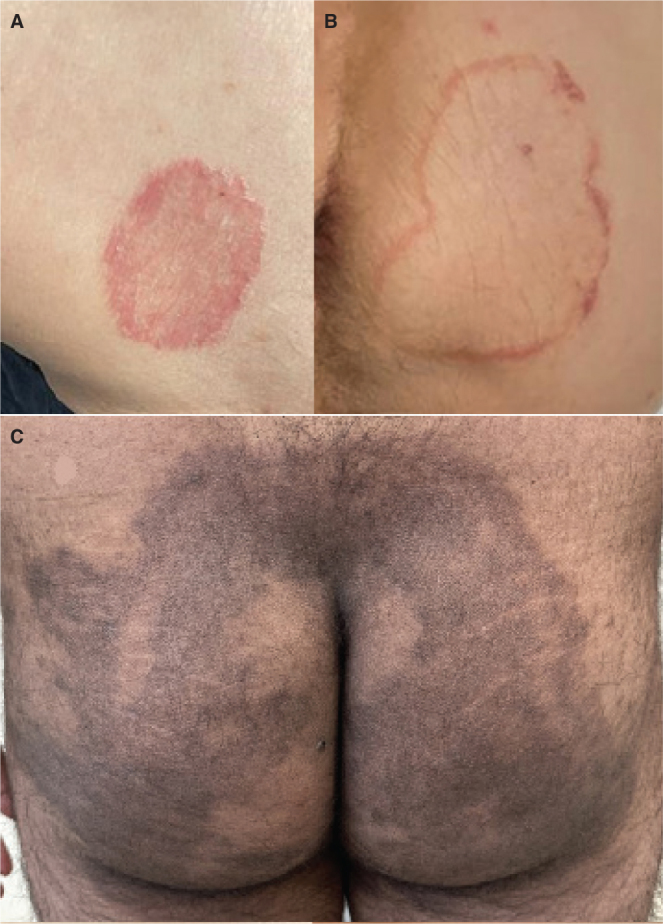
**Clinical images of lesions caused by terbinafine resistant *Trichophyton indotineae*.** Patient B (top left and right) patient C (bottom).

*Patient B.* In December 2022, a 68-year-old man with well-controlled HIV infection developed a sudden-onset lesion on his right buttock during a trip to the Canary Islands, Spain. Over time, more well-defined scaly lesions appeared on his thighs and glutes shown in Fig. 1 top left and right. Routine mycology cultures confirmed the diagnosis of dermatophytosis caused by *Trichophyton mentagrophytes* complex. Despite undergoing a 6-week course of oral terbinafine, there was no improvement in his condition. Subsequent antifungal susceptibility testing demonstrated terbinafine resistance (MIC >8 mg/mL). Consequently, oral itraconazole therapy was initiated at a dosage of 100 mg twice daily for 4 weeks, resulting in partial improvement. The treatment was extended for another 4 weeks, and after a total of 8 weeks of oral itraconazole at a dosage of 200 mg daily, the patient achieved complete clearance of the skin lesions. Interestingly, the patient’s partner, who also had well-controlled HIV, presented with the same infection and achieved resolution following an 8-week course of oral itraconazole therapy.

*Patient C.* In December 2021, a healthy 25-year-old man presented with a 7-month history of pruritus and plaque in the groin area extending towards the gluteal region. He denied any travel outside Europe in the year leading up to the appearance of his lesions. Fungal cultures confirmed the presence of *Trichophyton mentagrophytes* complex, and the patient underwent a total of 3 cycles of terbinafine treatment, with the longest duration lasting for 10 consecutive weeks at a dosage of 250 mg daily. Despite the extended treatment, the patient experienced only partial improvement. Subsequently, a new culture was sent for antifungal susceptibility testing, revealing sensitivity to itraconazole and resistance to terbinafine (MIC = 8 mg/L). Oral itraconazole therapy was initiated at a dosage of 100 mg daily for 4 weeks, resulting in partial improvement of the lesions. Recognizing the need for intensified treatment, the dosage was increased to 100 mg twice daily for an additional 6 weeks. This adjustment led to complete clearance of the lesions, with no recurrence observed at the 3-month follow-up. The isolate was later re-analyzed and identified as *T. indotineae.*

### Materials,methods, and results

Skin scrapings were cultured on selective fungal media and the isolated dermatophytes were initially identified by macro- and microscopic characteristics. The MIC testing of terbinafine and itraconazole was performed by the EUCAST reference method for the antifungal susceptibility testing of microconidia-forming dermatophytes E.Def 11.0 (7) at Karolinska University Laboratory. The isolates of cases A and C were identified at Statens Serum Institut by amplifying and sequencing of the squalene epoxidase (SQLE) gene, as previously described (5), which also characterized the F397L mutation associated with terbinafine resistance. The isolate of case B was identified by sequencing the ITS region using NGS technology in cooperation with the Public Health Agency of Sweden. MALDI-TOF was performed using the MSI-2 application for *T. indotineae* identification and the isolate of case B showed, for *T. indotineae*, specific peak 6845 Dalton (8).

## DISCUSSION

The exact cause of the emergence of *T. indotineae* remains unclear, but a leading theory suggests it may be linked to the unrestricted use of topical steroid and antifungal agents in India (1). The challenge not only lies in the species’ identification, where it is often mistakenly diagnosed as *T. mentagrophytes*, which typically responds well to terbinafine treatment, but also in the lack of knowledge concerning *T. indotineae* due to its relatively recent appearance in Sweden (1, 4). At Karolinska University Laboratory, 51 cases of *T. indotineae* infection were diagnosed from 2023 to December 2024. In 34 (67%) cases, the isolate was resistant to terbinafine, but all isolates were susceptible to itraconazole.

Establishing treatment protocols for treatment of *T. indotineae* infections is crucial (9, 10). Based on expert opinion, the recommended treatment for *T. indotineae* infections is oral itraconazole at 200 mg daily for 8 weeks. A 100 mg daily dose for 4 weeks may also work but has a higher risk of incomplete resolution (Table 1). Some *T. indotineae* strains show resistance to itraconazole.

**Table 1:** Response to itraconazole treatment in 3 patients with terbinafine-resistant *Trichophyton indotineae*

Patient	Age/Sex	Initial treatment	Final treatment	Treatment outcome
A	21/F	Terbinafine 250 mg twice daily	Itraconazole 100 mg/day	Complete resolution after 4 weeks, no recurrence after 6 months
B	68/M	Terbinafine 250 mg daily	Itraconazole 200 mg/day	Partial improvement after 4 weeks, complete resolution after 8 weeks
C	25/M	Terbinafine 250 mg daily	Itraconazole 200 mg/day	Complete resolution after a total of 10 weeks, no recurrence at 3-month follow-up

To curb its spread effectively, it is vital that clinicians, researchers, and public health authorities collaborate closely. Clinicians should be aware of the risk of *Trichophyton indotineae* infection in patients with extensive, treatment-resistant tinea corporis, especially in those with a history of international travel, although infection within Sweden is also occurring, as shown by the reported cases. Suspected cases should be communicated to the laboratory to increase the possibility of an accurate diagnosis. Improving diagnostic methodologies, alongside establishing evidence-based treatment protocols, is critical. Through these concerted efforts, we can enhance our management of this challenging fungal infection and significantly improve outcomes for patients worldwide (1, 4, 6, 11).
